# Genomically Selected Genes Associated with a High Rate of Egg Production in Puan Panjiang Black-Bone Chickens

**DOI:** 10.3390/ani15030363

**Published:** 2025-01-27

**Authors:** Xiaomeng Miao, Zhiying Huang, Jia Liu, Li Zhang, Yulong Feng, Yalan Zhang, Diyan Li, Zhonghua Ning

**Affiliations:** 1 National Engineering Laboratory for Animal Breeding, College of Animal Science and Technology, China Agricultural University, Beijing 100193, China; mxm19920404@126.com (X.M.);; 2 Institute of Animal Husbandry and Veterinary Medicine, Guizhou Academy of Agricultural Sciences, Guiyang 550005, China; 3 College of Animal Science, Shanxi Agricultural University, Taiyuan 030801, China; 4 Guizhou Province Livestock and Poultry Genetic Resources Management Station, Guizhou Provincial Department of Agriculture and Rural Affairs, Guiyang 550001, China; 5 School of Pharmacy, Chengdu University, Chengdu 610106, China

**Keywords:** population genomics, gene selection, transcriptome, egg production, chicken

## Abstract

Puan Panjiang black-bone chickens are renowned for their distinctive traits, deep black coloration, and high-quality protein content, making them a focus of genetic research due to their unique egg-laying abilities. In this study, 110 Puan Panjiang black-bone chickens were used to investigate the effects of natural and artificial selection influencing egg production. Whole-genome resequencing data from red junglefowl (RJF) and high-egg-production (HEP) and low-egg-production (LEP) groups of Puan Panjiang black-bone chickens revealed significant genetic variants associated with egg production traits. Additionally, transcriptome analysis of 47 samples from ovary stroma, small white follicles (SWFs), small yellow follicles (SYFs), and liver tissues from 6 HEP and 6 LEP groups identified differentially expressed genes. Notably, differences in egg production were linked to small yellow follicles rather than ovary stroma or SWFs. Key candidate genes, including *TRIM7*, *CASR*, *SPTBN5*, *GAL1*, *ZP1*, *IL4I1*, and *CCL19,* were identified as potential contributors to egg-laying performance. This study underscores the genetic diversity within this breed and provides valuable insights for future breeding programs to enhance egg production, supporting the sustainable development of this local resource.

## Simple Summary

Puan Panjiang black-bone chickens are a native breed from southwest China. We aimed to explore the genetic basis underlying their egg production traits. Whole-genome resequencing and differential gene expression analysis were used to identify potential key genes and pathways influencing variations in egg-laying performance. These findings offer novel insights and serve as a valuable reference for future research aimed at improving egg production in hens.

## 1. Introduction

The Puan Panjiang black-bone chicken, also known as the Puan Niujiaoshan black-bone chicken, is a distinguished specialty from Guizhou, China, recognized as a marked product by the Ministry of Agriculture [1]. With over 2000 years of breeding history, this unique breed stands out as one of China’s rarest poultry breeds and one of the rarest birds in the world. Its distinct characteristics include a black crown, feathers, skin, meat, and bones, earning it the local nickname “black phoenix of the mountains” [2]. The chicken’s firm meat, well-proportioned body, flat head, single crown, predominantly black feathers with a touch of jute color, and well-developed tail feathers of the rooster contribute to its fame and popularity among enthusiasts. Puan Panjiang black-bone chickens are highly valued for their nutritional and health benefits [3]. The meat is rich in essential amino acids and melanin, which are believed to support liver function and kidney health. Its lean composition, characterized by low fat and high protein content, aligns well with modern dietary preferences, making it an appealing choice for contemporary diets. The poultry quality metrics further highlight its superior nutritional profile, with a protein content exceeding 23.5 g/100 g, a fat content at or below 3.0 g/100 g, a comprehensive amino acid profile above 17.0 g/100 g, and a moisture content below 74.0 g/100 g [4].

The laying performance of hens is of great economic importance in the poultry industry, and egg production is a polygenic trait with low to moderate heritability [5]. As an agricultural animal, chicken is an important type of poultry, providing a major source of both meat and eggs. Eggs are a high-quality source of protein for humans and are an essential human food widely consumed worldwide because of their high digestibility and balanced amino acid composition [6]. Egg production is affected by several factors. For example, the main endocrine factors that regulate egg laying are gonadotropin-releasing hormone, prolactin, follicle-stimulating hormone, and luteinizing hormone [7]. The expression of *ACSF1* is upregulated by estrogen via ERα, and the expression of *ACSF2* is downregulated by estrogen, which might be related to egg-laying performance in chickens [8]. The *MMP13* gene in Chahua chickens is important for egg-laying performance [9]. Genome sequence comparison is a modern extension of the long-standing use of livestock breeding [10].

In the modern poultry industry, enhancing egg production in chickens has been a focal point of genetic selection research. With the rapid advancements in genomics and bioinformatics technologies, genome-wide association studies (GWASs) have become crucial tools for understanding the genetic basis of high-egg-production traits in chickens [11]. Recent studies utilizing GWASs have identified key single-nucleotide polymorphisms (SNPs) and candidate genes affecting chicken egg production traits, offering valuable insights for further molecular breeding efforts [12,13]. One study identified 148 SNPs associated with egg number traits by comparing high- and low-egg-production groups, and through functional annotation, 32 candidate genes, such as *NELL2*, *KITLG*, and *GHRHR*, which were previously proven to be related to egg production and reproductive traits, were identified. Additionally, this research highlighted a significant peak on chromosome 5, showing significant allele frequency differences for specific SNPs associated with egg production differences [14]. Another comprehensive study analyzed egg production performance throughout the laying period, identifying 161 unique candidate SNPs across multiple chromosomes. Association tests using a univariate linear mixed model linked these SNPs to the age at first egg (AFE) and egg number (EN) at different stages. This study emphasized the importance of certain genes, such as *GATA3* and *HIPK3*, for understanding the molecular mechanisms behind egg production traits [15]. Finally, research focused on maternal lines of meat-type chickens revealed that a significant quantitative trait locus (QTL) on chromosome Z affects egg production traits [16]. Genetic parameter analyses across different lines revealed the low heritability of egg number traits, suggesting that diverse genetic backgrounds or environmental effects influence this complex trait. The ovary and liver are important tissues for chicken egg production [17]. Recent studies revealed that there are 13 distinct cell types in chicken ovaries, and the majority of the differentially expressed genes presented higher expression levels in Lohmann chickens with relatively high egg production [18]. However, no study has focused on different parts or follicle developmental stages in the ovaries of chickens with different egg production rates.

This study aimed to explore the genetic factors underlying egg production in Puan Panjiang black-bone chickens. By integrating whole-genome resequencing and transcriptome analysis, the research sought to identify genetic variants and differentially expressed genes associated with egg-laying performance. These findings will deepen the understanding of the genetic diversity and biological mechanisms regulating egg production, thereby contributing to the conservation and sustainable utilization of Puan Panjiang black-bone chickens.

## 2. Materials and Methods

### 2.1. Chickens

**Figure 1:**
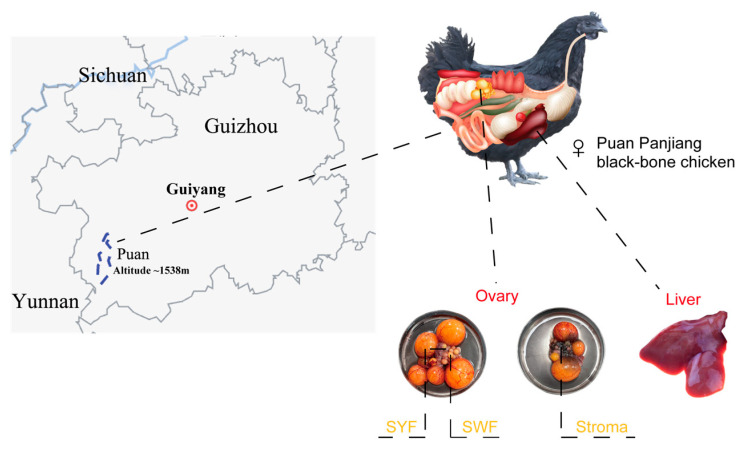
The geographical location and sampling locations of Pu’an County, Guizhou Province, China.

A total of 1288 female Puan Panjiang black-bone chickens hatched on the same day were selected and reared at Guizhou Jinhe Poultry Industry Co., Ltd. in Qianxinan, China. The chickens were fed according to standard on-farm feeding practices and watered ad libitum. To select high-egg-production (HEP) and low-egg-production (LEP) groups of chickens with similar body weight in the population, the egg production of each chicken was recorded from onset of laying to peak (33 weeks). The Wilcoxon rank-sum test (Mann–Whitney U test) was applied to evaluate the statistical significance between the two groups. For genome resequencing, we collected 110 blood samples (55 from HEP chickens and 55 from LEP chickens) via the wing vein. These sample genomes were used for comparative genome analysis together with 17 red junglefowl (RJF) genomes downloaded from NCBI. Six high-egg-production chickens and six low-egg-production chickens were cervically dislocated and humanely slaughtered before fresh tissues, including SWF, SYF, stroma, and liver samples, were collected, and each sample was taken in duplicate. All sample collection procedures were conducted aseptically on a clean bench. Samples were immediately snap-frozen in liquid nitrogen after collection, transported to the laboratory, and stored at −80 °C until subsequent transcriptome analysis. The sample collection overview is presented in Figure 1 and Table 1.

### 2.2. Genomic DNA Extraction

Total DNA was isolated from blood using the TIANamp Genomic DNA Kit (Tiangen Biotech) according to the manufacturer’s instructions. The DNA concentration was quantified with a NanoDrop2000 spectrophotometer, and its integrity was evaluated using 1% agarose gel electrophoresis.

### 2.3. Whole-Genome Sequencing and Quality Control

After verifying the quality of the DNA samples, random fragmentation was carried out using an ultrasonicator (Covaris Inc., Woburn, MA, USA). A sequencing library was prepared with the NEBNext^®^ Ultra^™^ DNA Library Prep Kit for Illumina (NEB, Ipswich, MA, USA) following the manufacturer’s instructions. Indexes were incorporated to uniquely tag each sample. The library preparation process involved end repair, dA tailing, and ligation with a full-length adapter for sequencing, followed by PCR amplification and purification. The resulting DNA libraries, with an insert size of 350 bp, were constructed. Genomes from 110 individuals were sequenced individually using 150 bp paired-end reads on the DNBSEQ-T7 platform (Novogene Bioinformatics Technology Co., Ltd., Beijing, China). To minimize sequencing errors and reduce noise in the analysis, low-quality paired reads were excluded using an in-house script. The exclusion criteria consisted of ≥ 10% unidentified nucleotides (N), >10 nucleotides aligned to the adapter (allowing ≤ 10% mismatches), >50% bases with Phred quality < 5, and putative PCR duplicates generated during library construction [19]. As a result, 2.52 terabases (approximately 21.76-fold coverage per individual) of high-quality paired-end reads were obtained, with 95.79% of nucleotides having Phred quality ≥ Q30 (accuracy of 98.90%).

### 2.4. Read Mapping, Genomic Variant Calling, and Annotation

The high-quality reads from each individual were aligned to the reference chicken genome (GRCg7b) using the Burrows–Wheeler Alignment tool (BWA, version 0.7.15) [20] with the ‘mem -t 10 -k 32’ command. The alignment process generated BAM files using SAMtools (version 0.1.19) [20]. To enhance alignment accuracy, we applied filters to retain reads with mismatches ≤ 5 and mapping quality ≥ 30. After sorting the initial BAM file with SAMtools, duplicate reads were marked using the “MarkDuplicates” command from the Picard package (version 1.119). Next, gVCF calling was performed using the Genome Analysis Toolkit (GATK) best practices pipeline (version 3.7) [21], employing the HaplotypeCaller method. Population SNP calling was conducted by merging all gVCFs using the “CombineGVCFs” command. To ensure SNP reliability, stringent variant filtration criteria were applied to exclude potential false positives: (a) quality by depth > 10.0; (b) mapping quality score > 40.0; (c) FS < 60.0; (d) MQRank-Sum > −12.5; (e) ReadPosRankSum > −8.0. SNPs with adjacent distances ≤ 5 were also excluded [22]. For final selection, high-credibility SNPs were filtered using vcftools (version 0.1.15) with the following parameters: sample call rate > 90%, SNP call ratio > 95%, minor allele frequencies > 1%, and Hardy–Weinberg equilibrium *p* value < 10^−5^. This filtering process resulted in 15.25 million and 15.28 million high-credibility SNPs in high- and low-egg-production chickens, respectively. These SNPs were categorized into various genomic regions (exonic, intronic, splice site, upstream and downstream gene regions, and intergenic). The ANNOVAR package was used for annotation [23].

### 2.5. Calculation of θπ and Fst

The selective sweep analysis incorporates the sequence diversity statistic (*θπ*) and the population differentiation statistic (*Fst*) to identify genomic regions under selection in two populations [24]. A sliding-window approach (40 kb windows sliding in 10 kb steps) was used to quantify polymorphism (*θπ*, pairwise nucleotide variation) and genetic differentiation (*Fst*) between RJF and Puan Panjiang black-bone chickens. The PopGenome package was used to calculate the *Fst* and *θπ* ratio of the SNPs within each window [25]. When two groups were compared, regions with significant differences (*p* < 10^−16^, Mann–Whitney U test) in the log2(*θπ* ratio) and *Fst* values (top 5%) were identified as selective regions.

### 2.6. Total RNA-Seq and Data Analysis

Total RNA was extracted from tissue samples (ovaries, liver, and stroma) using the Qiagen RNeasy Kit (Qiagen, Hilden, Germany). Sequencing libraries were prepared from 47 samples using the NEBNext Ultra RNA Library Prep Kit for Illumina (NEB, USA, Catalog #: E7530L). mRNA was isolated using poly-T oligo-attached magnetic beads according to the manufacturer’s protocol. Paired-end sequencing (2 × 150 bp) was performed on the DNBSEQ-T7 platform. The high-quality reads were mapped to the chicken reference genome using TopHat2 (v2.0.14) [26]. Aligned reads were assembled with StringTie (v1.3.3), and transcript construction was performed using Cufflinks (v2.0.2) [27,28]. Transcript expression levels were quantified as transcripts per million (TPM) using StringTie. Transcripts with TPM ≥ 0.5 in at least two biological replicates were considered expressed protein-coding genes (PCGs). Differential expression analysis of PCGs was conducted using DESeq2 (v1.20.0) [29]. Gene ontology enrichment analysis of differentially expressed genes was performed using Metascape (https://metascape.org) [30,31].

## 3. Results

### 3.1. Data Summary and Variation Discovery

**Figure 2:**
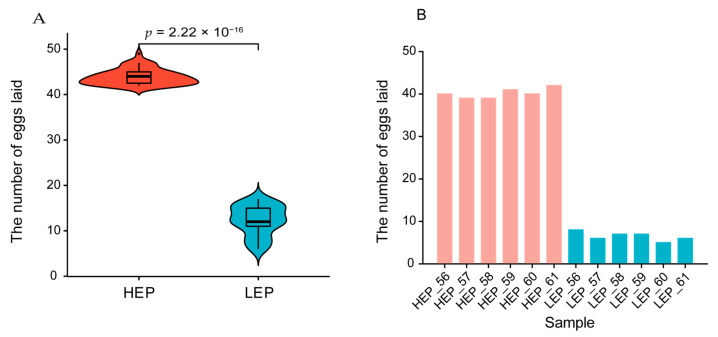
The comparative egg production performance of HEP and LEP. (**A**) Violin plot comparing egg production in the HEP and LEP groups. Significant differences in egg production were observed, with HEP laying more eggs than LEP (*p* = 2.22 × 10^−16^). (**B**) Bar graph showing egg production for each sample in the HEP and LEP groups.

In this study, the number of eggs laid by each hen was recorded, and the egg production for the HEP and LEP groups was displayed in Figure 2. Figure 2A highlights a highly significant difference in egg production between the HEP and LEP groups, while Figure 2B illustrates the egg production of individual samples from each group used for transcriptome sequencing. We sequenced 110 black-bone chickens from Puan County, Guizhou Province, and performed transcriptome sequencing on the liver and ovary tissues of 12 black-bone chickens, obtaining approximately 2.52 trillion bases (Tb) of genome resequencing data. After filtering, we aligned the reads to the reference chicken genome, achieving approximately 21.76-fold coverage per individual (Table S1). Additionally, we included previously published genome sequence data from 17 red jungle fowl (RJF) with an average coverage of approximately 23-fold per individual obtained from downloaded and analyzed datasets (GenBank accession numbers are provided in Table S1). Upon aligning this extensive dataset to the reference chicken genome, we identified approximately 15.25 million and 15.28 million single-nucleotide polymorphisms (SNPs) and 16.09 million and 16.10 million insertions and deletions (InDels) for the HEP and LEP groups, respectively. The categorization of SNPs for the HEP and LEP groups is presented in Table S2. For a comprehensive exploration of mRNA expression profiles across various tissues and breeds in chickens, we constructed a total of 47 cDNA libraries. These libraries included samples from 12 SWF, 11 SYF, 12 liver, and 12 stroma tissues, all of which were obtained from 12 female chickens. The dataset included 318.68 gigabases (Gb) of clean data, with an average of 6.78 Gb per sample (Table S3).

### 3.2. Genome-Wide Selective Sweep Signals in Puan Panjiang Black-Bone Chickens

**Figure 3:**
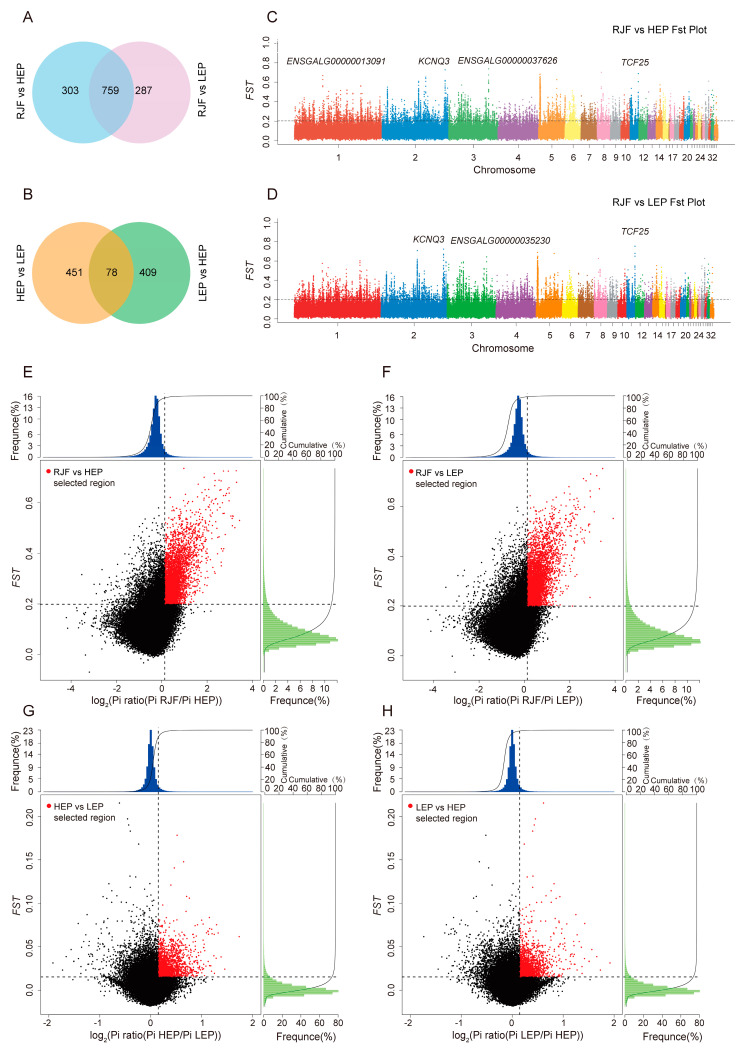
Genomic regions displaying robust selective sweep signals were examined in Guizhou local chicken breeds and RJFs. Venn diagrams showing the number of selection genes identified by comparing RJF and the two chicken populations separately (**A**) and when the two populations are compared with each other (**B**). Genome-wide selective scanning analysis between HEP (**C**) and LEP (**D**) and RJF. Horizontal dashed lines show the significance level of α = 0.05. The distribution of θπ ratios (*θπ*, domestic/*θπ*, RJF) and *FST* values for HEP and LEP (**E**–**H**) is presented, calculated within 40 kb windows sliding in 10 kb increments. Data points situated to the left and right of the left and right vertical dashed lines, respectively (corresponding to the 5% left and right tails of the empirical *θπ* ratio distribution) and above the horizontal dashed line (representing the 5% right tail of the empirical *FST* distribution), were identified as selected regions for HEP and LEP (depicted as red points), respectively.

The selective sweep screen employs a method that incorporates the sequence diversity statistic (*θπ*) and the population differentiation statistic (*Fst*) [32,33]. This methodology was specifically designed to identify genomic regions under selection in two populations of Puan Panjiang black-bone chickens. Through our analyses, we identified 1062 and 1046 positively selected genes (PSGs) in the HEP and LEP groups versus RJF, respectively (Figure 3A), and we identified 529 and 487 PSGs in the HEP and LEP intercomparison, respectively (Figure 3B), indicating strong selective sweep signals in each population. In both comparisons, we identified 759 and 78 genes that exhibited strong selective scanning signals. The selected regions, characterized by significantly high *Fst* values (top 5%), were displayed for HEP (Figure 3C) and LEP (Figure 3D) chickens compared with RJFs. Regions showing significant differences (*p* < 10^−16^, Mann–Whitney U test) in the log2(*θπ* ratio) and *Fst* values compared with the wild RJF genomic background are highlighted for both the HEP (Figure 3E) and LEP (Figure 3F) chicken populations. When the two groups were compared, regions showing significant differences were highlighted in the HEP (Figure 3G) and LEP (Figure 3H) chicken populations.

Notably, compared with the wild-type RJF genomic background, 35 and 47 regions presented *Fst* values greater than 0.5 for HEP and LEP chickens, respectively. The top ten selected genes according to *Fst* size are shown in Table 2. Within these selected regions in HEP chickens, 27 known genes were identified, including *KCNQ3, PLAA, TCF25, PSD3, MOG, SSBP2, TGA1, CAAP1, BECN1, PSME3, KDM4C, MC1R, NRG1, SMG1, KIF18A, METTL15P1, TSNARE1, TSHR, DAGLB, RAC1, LIFR, PELO, CNTD1, GADD45G, APP, BDNF*, and *DDX27*. Similarly, 32 known genes were identified in LEP chickens, including *TCF25, PSD3, KCNQ3, PLAA, MC1R, BECN1, PSME3, CAAP1, VSTM2A, TSNARE1, LIFR, ITGA1, NCBP1, KIZ, FANCC, LRRC19, NRG1, KDM4C, COQ7, TMC7, ROR2, GPBP1, WDR70, CELF4, TMEM132C, CNTD1, SSBP2, KIF18A, DAPK1, BDNF, SMG1,* and *DEF8*, within its selected regions.

### 3.3. Signatures of Selection in the Two Chicken Populations

**Figure 4:**
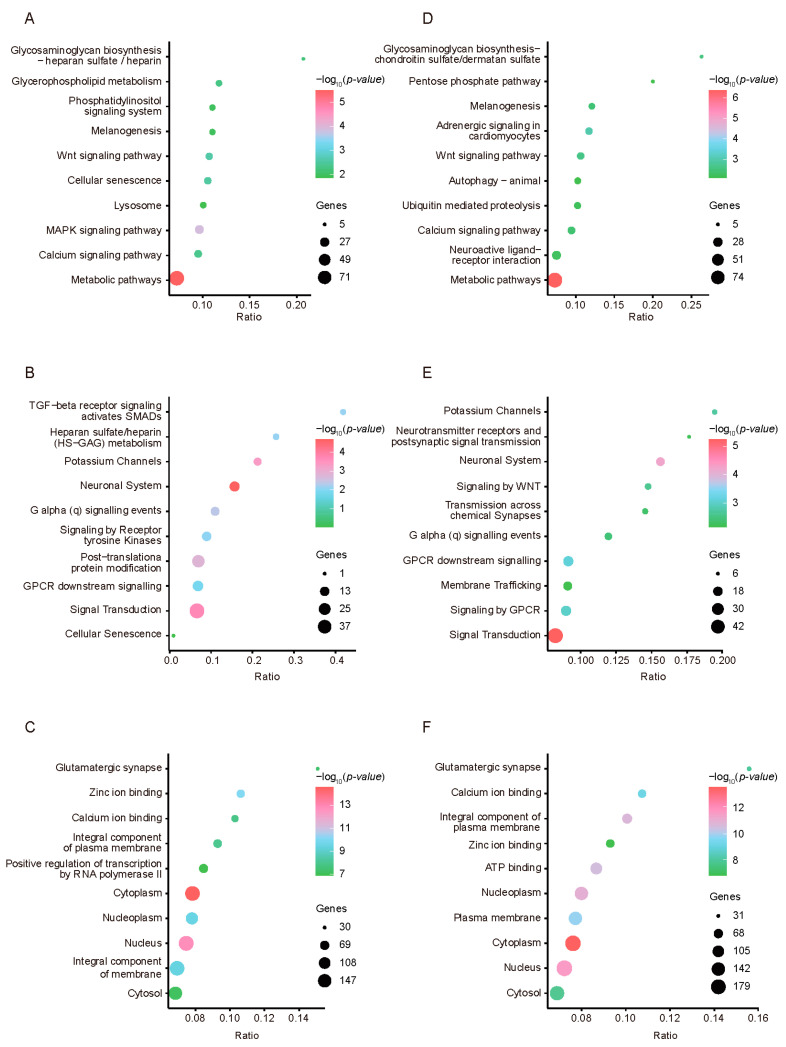
Enrichment analysis of selected genes in two chicken populations. KEGG, Reactome, and GO analyses of selected genes in HEP (**A**–**C**) and LEP (**D**–**F**) in comparison with RJF are shown.

To explore the potential functions of the identified genes in the two chicken breeds, we performed gene ontology enrichment using Metascape. Compared with RJF, the KEGG classification analysis results of the HEP group indicated that the high-yield layer group was enriched in pathways such as “glycosaminoglycan biosynthesis heparan sulfate/heparin”, “glycerophospholipid metabolism”, and “phosphatidylinositol signaling system” (Figure 4A). These findings suggest that high-egg-production hens may have significantly evolved metabolically through artificial selection to adapt to high energy and nutritional demands. The REACTOME classification primarily highlighted the enrichment of biological functions related to “TGF-beta receptor signaling activates SMADs”, “heparan sulfate/heparin (HS-GAG) metabolism”, “potassium channels”, and “GPCR downstream signaling” (Figure 4B). These pathways play crucial roles in cellular signal transduction and regulation, possibly related to the protein synthesis and metabolic activities of high-yielding layers. The adjustments in growth and development control highlighted may be the result of selection aimed at optimizing production performance. Gene ontology emphasized pathways such as “glutamatergic synapse”, “calcium ion binding”, and “zinc ion binding” (Figure 4C). These results reveal the importance of intracellular ion balance and signaling in maintaining the physiological state of high-yield layers. Moreover, the KEGG classification analysis results for the LEP group highlighted pathways such as “glycosaminoglycan biosynthesis chondroitin sulfate/dermatan sulfate”, “pentose phosphate pathway”, and “ubiquitin mediated proteolysis” (Figure 4D). These findings suggest that certain metabolic pathways might have been under strong selective pressure during evolution, leading to low egg-production performance in chickens. The REACTOME classification emphasized pathways related to “potassium channels”, “neurotransmitter receptors and postsynaptic signal transmission”, and “neuronal system” (Figure 4E). These findings indicate that in the selection process, the production performance of low-yielding layers might be directly related to the regulation and signaling mechanisms of the nervous system. Gene ontology classification revealed enrichment in pathways such as “glutamatergic synapse”, “calcium ion binding”, and “ATP binding” (Figure 4F). These results may reflect specific adaptations in cellular structure and function in low-yield layers.

**Figure 5:**
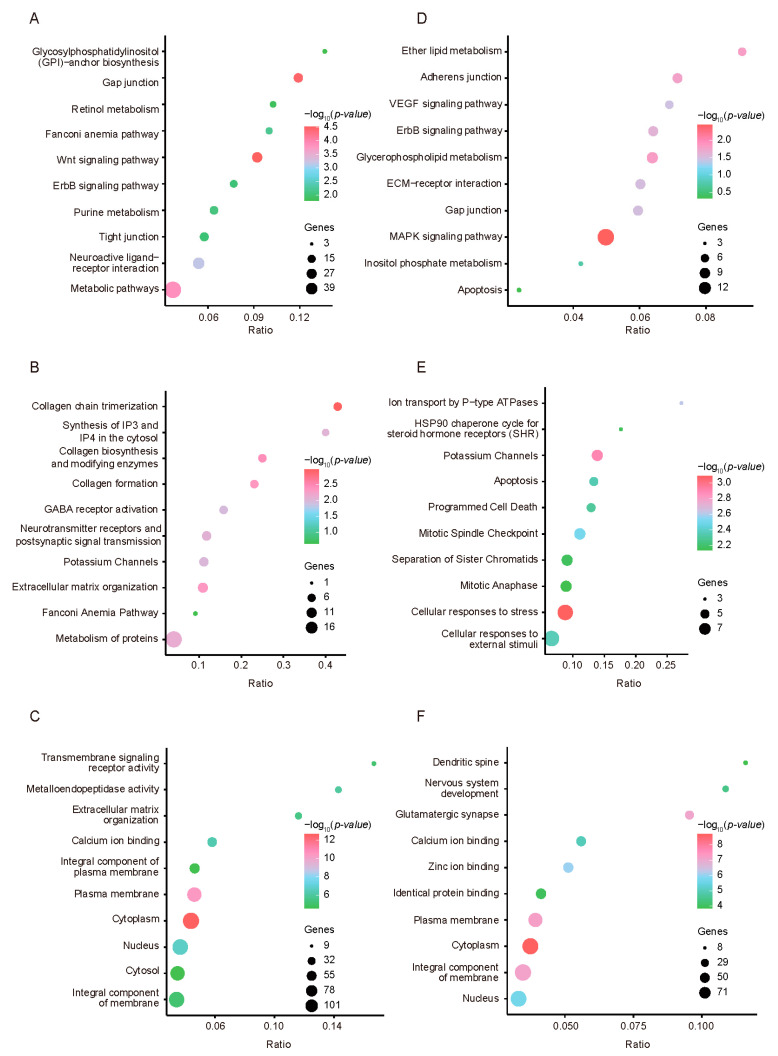
Enrichment analysis of selected genes in two chicken populations. KEGG, Reactome, and GO analyses of selected genes in HEP (**A**–**C**) and LEP (**D**–**F**) in two chicken populations compared with each other are shown.

Compared with those of the LEP group, the KEGG classifications of the HEP group highlighted key biological pathways such as “Glycosylphosphatidylinositol (GPI)-anchor biosynthesis”, “Retinol metabolism”, and “Wnt signaling pathway” (Figure 5A). These pathways might be particularly active in the HEP population, which is related to high egg-production performance. Reactome analysis focused on pathways such as “collagen biosynthesis and modifying enzymes”, “collagen chain trimerization”, “GABA receptor activation”, and “neurotransmitter receptors and postsynaptic signal transmission” (Figure 5B), reflecting potentially optimized biological functions in HEP chickens for maintaining cellular structure and neural transmission. The GO analysis emphasized the organizational function of the cell membrane and extracellular matrix, as well as the activity of calcium ion binding, involving pathways such as “transmembrane signaling receptor activity”, “extracellular matrix organization”, “calcium ion binding”, and “metalloendopeptidase activity” (Figure 5C). This may be the biological basis for high-yield layers adapting to high production demands. Conversely, the KEGG classification for the LEP group revealed active pathways such as “ether lipid metabolism”, “VEGF signaling pathway”, and “ErbB signaling pathway” (Figure 5D), which may indicate differences in cellular growth and metabolic regulation compared with HEP chickens. Reactome analysis revealed pathways such as “Ion transport by P-type ATPases” and “HSP90 chaperone cycle for steroid hormone receptors (SHRs)” (Figure 5E), suggesting that LEP chickens might have different regulatory mechanisms for cell cycle control and stress response compared with HEP chickens. The GO analysis focused on the development of the nervous system and the function of glutamatergic synapses, including pathways such as “glutamatergic synapse”, “nervous system development”, and “dendritic spine” (Figure 5F), indicating that LEP chickens might maintain different adaptabilities in neural regulation and cellular function.

### 3.4. Analysis of Differentially Expressed Genes

**Figure 6:**
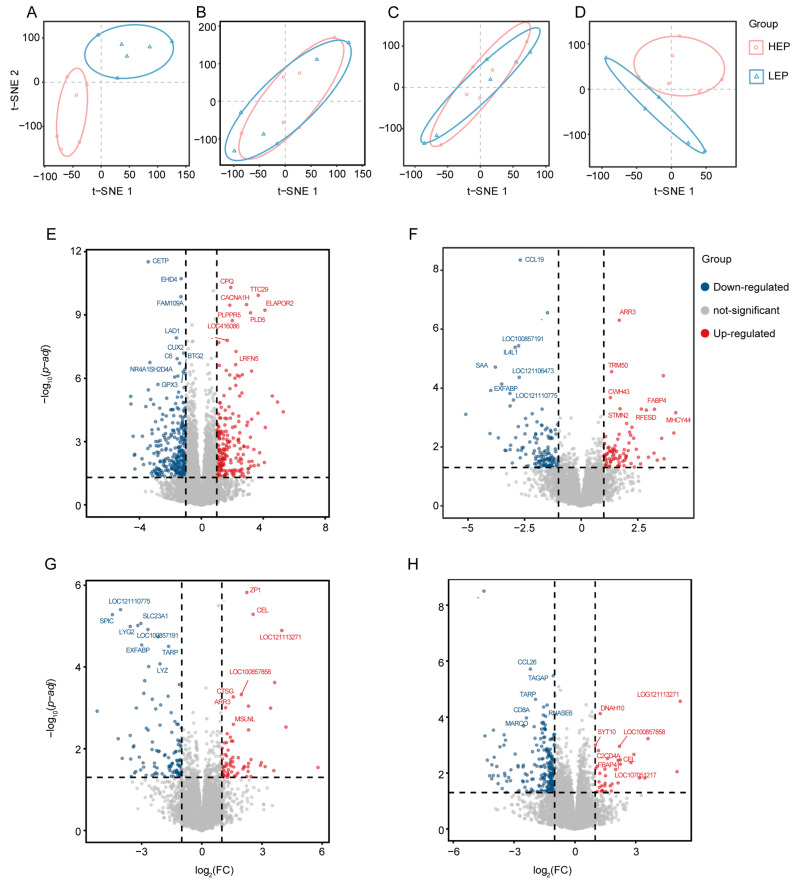
Analysis of differentially expressed genes. (**A**–**D**) Principal component analysis (PCA) plots of liver (**A**), stroma (**B**), SWF (**C**), and SYF (**D**) between HEP and LEP. (**E**–**H**) Volcano plots of differentially expressed genes in the liver (**E**), stroma (**F**), SWF (**G**), and SYF (**H**) between HEP and LEP. The horizontal axis reflects the fold change distribution of the differentially expressed genes, typically represented as Log2(fold change). The further a point deviates from the center, the greater the fold change. The vertical axis is −Log10(adjusted *p*-value), with points closer to the top and bottom indicating higher significance. In the plot, red points represent upregulated genes, blue points represent downregulated genes, and gray points indicate genes with no significant difference.

Differentially expressed gene (DEG) analysis was performed to explore the differences between HEP and LEP chickens in four different tissues, utilizing TPM for gene expression quantification. The PCA graph revealed that the liver (Figure 6A) is the main site for yolk and protein synthesis. t-SNE analysis revealed that the HEP and LEP liver samples were somewhat separated, indicating that there may be significant differences in the expression of genes related to liver function between the two groups. These differences may be related to the efficiency and quality of yolk synthesis, which directly affect the yield and nutritional value of eggs. The ovarian stroma provides the necessary structural and nutritional support for the oocytes in the ovary. The vascular and cellular environment here is crucial for oocyte maturation and the synthesis and regulation of hormones, affecting oocyte quality and development. Small white follicles constitute the primary follicle stage in the ovary. These follicles contain immature oocytes, which gradually mature and eventually develop into oocytes capable of ovulation. Gene expression and hormone regulation at this stage are crucial for the maturation of follicles and directly affect the formation and accumulation of yolk. However, in the ovarian stroma (Figure 6B) and SWF (Figure 6C), the sample clustering was relatively tight. This finding reflects that the degree to which the two groups affect the health and maturation of the oocytes in terms of the ovarian environment and hormone regulation is not obvious. Finally, small yellow follicles are the more developed follicle stage in the ovary, and they play a decisive role in yolk accumulation. At this stage, lipids and other nutrients accumulate around the egg cells in preparation for the final yolk formation. In addition, small yellow follicles are also a stage where hormone activity is particularly active, and hormone regulation is essential for the further development of the follicles and the determination of ovulation time. In the clustering of small yellow follicles (Figure 6D), there was a significant separation between HEP and LEP samples, and the transcriptome expression profiles of the two groups were very different at the critical stage of yolk accumulation. These findings revealed that there are key genes that affect the difference in egg production between the two groups at this stage.

A comparison of HEP and LEP groups revealed 494 differentially expressed genes (DEGs) in the liver, including 294 downregulated genes and 200 upregulated genes (Figure 6E). In the stroma, there were 212 DEGs, including 132 downregulated genes and 80 upregulated genes (Figure 6F). In SWF, there were 172 DEGs, including 106 downregulated genes and 66 upregulated genes (Figure 6G). In SYF, there were 250 DEGs, including 214 downregulated genes and 36 upregulated genes (Figure 6H). For stroma, SWF, and SYF, SYF had more DEGs (SWF: 172; stroma: 212; SYF: 250), indicating the important role of SYF in the follicle selection process, which involves the subtle fine-tuning of the expression levels of many genes. The top five expressed genes in each tissue according to the absolute value of log2FC are shown in Table 3.

**Figure 7:**
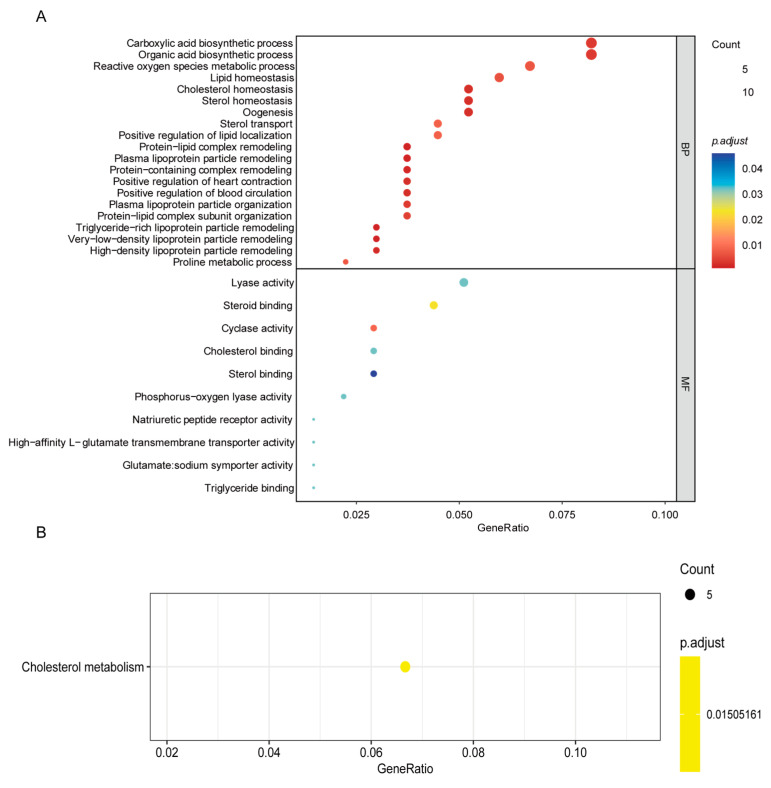
Functional enrichment analysis of differentially expressed genes in liver samples. (**A**) Gene ontology (GO) of differentially expressed genes in liver samples from the HEP group compared to the LEP group. (**B**) KEGG enrichment analysis of differentially expressed genes in liver samples from the HEP group compared to the LEP group.

The functional enrichment analysis of the differentially expressed genes in these tissues revealed that, compared with those in LEP livers, the differentially expressed genes in HEP livers are associated primarily with biological processes such as “lipid homeostasis”, “cholesterol homeostasis”, “lipoprotein particle remodeling”, and “lipoprotein particle organization” (Figure 7A). The processes of lipid and cholesterol homeostasis and transport are essential for maintaining the integrity and function of the cell membrane, particularly during yolk formation. The lipids within the yolk serve as the main energy source for embryonic development. Lipoprotein particles in the plasma are responsible for the transport of lipids, including cholesterol and triglycerides. Alterations in their structure and function may affect the efficiency of lipid transport, thereby impacting energy storage and utilization, which are particularly crucial for the energy-demanding egg-laying process. Therefore, the ability to regulate the synthesis, translocation, and redistribution of these substances may directly influence egg size, quantity, and quality. Furthermore, these genes are related to molecular functions such as “steroid binding”, “cholesterol binding”, “sterol binding”, and “lyase and cyclase activities” (Figure 7A). The binding activities of steroids and cholesterol reflect the cell’s responsiveness to these molecules, with steroids such as sex hormones playing significant roles in regulating the egg-laying cycle. These enzymes are involved in various biosynthetic pathways, including the production of sterols and other key biomolecules. Their activity may affect the metabolic efficiency and biosynthetic capacity of cells, which is essential for maintaining a high-capacity metabolic state. Thus, the effective binding and transport capabilities of steroids and cholesterol may affect the hormonal balance and, consequently, the egg-laying performance. Additionally, the KEGG enrichment analysis clearly revealed enrichment of the “cholesterol metabolism” pathway (Figure 7B). Cholesterol serves as a precursor for many hormones, including sex hormones, which regulate egg laying. The efficiency and direction of cholesterol metabolism may impact hormone levels, thereby indirectly affecting the capacity for egg laying.

**Figure 8:**
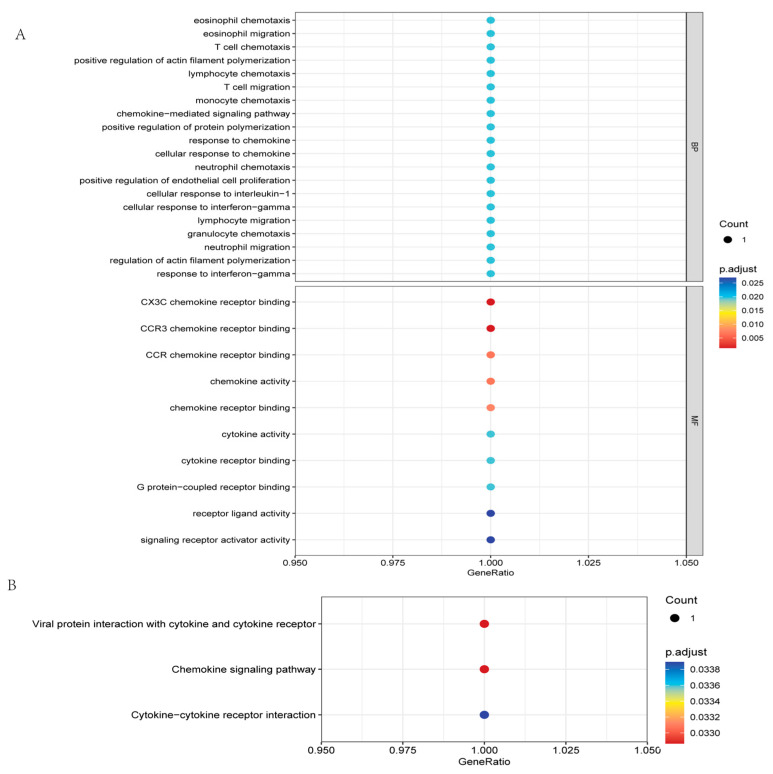
Functional enrichment analysis of differentially expressed genes in SYF samples. (**A**) Gene ontology (GO) of differentially expressed genes in SYF samples from the HEP group compared to the LEP group. (**B**) KEGG enrichment analysis of differentially expressed genes in SYF samples from the HEP group compared to the LEP group.

In the SYF, gene ontology enrichment analysis revealed that the differentially expressed genes were associated mainly with biological processes and molecular functions such as “cellular response to interferon-gamma”, “cytokine activity”, and “cytokine receptor binding” (Figure 8A). KEGG pathway analysis revealed the involvement of pathways such as the “chemokine signaling pathway” (Figure 8B). The activity of these pathways might indicate differences in immune regulation and cellular signaling between the two chicken groups, which could be one of the reasons for the differences in egg production between the groups. In particular, the strength and regulation of immune responses may directly affect the distribution and utilization of energy, thereby impacting egg production performance. Healthy chickens are better able to resist diseases and maintain high levels of production performance, whereas an overly active or dysfunctional immune system might consume excessive energy, affecting production performance. These analyses involve the chicken immune response and cellular signal transduction, which can directly or indirectly influence the health and physiological status of chickens, potentially affecting egg production performance.

## 4. Discussion

Egg-laying performance is one of the most important reproductive performances of hens and plays an important role in the poultry industry [34]. Puan Panjiang black-bone chickens, a specialty of the Qianxinan region in Guizhou, China, have been recognized as a signature agricultural product by the Ministry of Agriculture [35]. These chickens are one of the most representative and distinctive local products in Guizhou and across the country. The unique geographical and cultural conditions of southwest Guizhou have given Puan Panjiang black chickens a unique status and fame, making them highly favored among enthusiasts [3]. Therefore, research on the genomic breeding genes related to high egg-production rates in Puan Panjiang black-bone chickens is crucial. During animal domestication, both natural and artificial selection processes have significant impacts on the genome [36,37,38]. Rapidly advancing sequencing technologies have facilitated the study of genetic variations associated with different phenotypes [39]. In this study, we conducted a selection sweep analysis using SNPs and Indels from the whole-genome sequencing of 110 chicken samples, identifying genomic breeding genes associated with high egg-production rates in Puan Panjiang black-bone chickens.

In this study, the genes with the highest levels of selection in HEP chickens included *KCNQ3*, *PLAA*, *TCF25*, *PSD3*, *MOG*, *SSBP2*, *TGA1*, *CAAP1*, *BECN1*, *PSME3*, *KDM4C*, *MC1R*, *NRG1*, *SMG1*, *KIF18A*, *METTL15P1*, *TSNARE1*, *TSHR*, *DAGLB*, *RAC1*, *LIFR*, *PELO*, *CNTD1*, *GADD45G*, *APP*, *BDNF,* and *DDX27*. Similarly, 32 known genes were revealed in LEP chickens, including *TCF25*, *PSD3*, *KCNQ3*, *PLAA*, *MC1R*, *BECN1*, *PSME3*, *CAAP1*, *VSTM2A*, *TSNARE1*, *LIFR*, *ITGA1*, *NCBP1*, *KIZ*, *FANCC*, *LRRC19*, *NRG1*, *KDM4C*, *COQ7*, *TMC7*, *ROR2*, *GPBP1*, *WDR70*, *CELF4*, *TMEM132C*, *CNTD1*, *SSBP2*, *KIF18A*, *DAPK1*, *BDNF*, *SMG1,* and *DEF8*, within its selected regions. Among these genes, *PSD3* can realize guanylate-nucleotide exchange factor activity and phospholipid binding activity. Dietmar identified two to five novel potential tumor-suppressor or antagonist gene candidates in ovarian cancer from chromosome band 8p22 (N33 and EFA6R, with effects on survival, and potentially FLJ32642, MTSG1, and PCM1), which are promising candidates for further functional analysis of ovarian cancer [40]. Common variants in *PSD3* are associated with high-density lipoprotein cholesterol levels [41]. *BECN1* encodes a protein that regulates autophagy and is a component of the phosphatidylinositol 3-kinase (PI3K) complex, which mediates vesicle trafficking and may affect the health and function of germ cells [42,43]. The protein encoded by the *DDX27* gene is a putative ATP-dependent RNA helicase that is part of the ribosomal RNA (rRNA) processing machinery in the nucleolus and is specifically involved in regulating the formation of the 3′ end of the ribosomal 47S rRNA [44]. Considering its role in ribosome biogenesis, we anticipate that it may indirectly affect biological processes that are highly dependent on protein synthesis, such as oocyte maturation and yolk formation in the ovary. The protein encoded by the *TSHR* gene is a membrane protein that is a major controller of thyroid cell metabolism. The encoded protein is a receptor for thyroid hormone and thyroid-stimulating hormone, and its activity is mediated by adenylate cyclase [45,46]. Adjustments in thyroid hormone levels can affect an animal’s reproductive cycle and reproductive health [47]. We speculate that these genes may affect the development of egg-laying phenotypes in chickens; however, their exact mechanisms of action remain unclear.

To further explore the adaptive changes in chicken breeds during natural and artificial selection, we performed transcriptome sequencing on 47 samples to identify DEGs in tissues such as the liver and ovary of two high- and low-yield chicken populations and to gain an in-depth understanding of the molecular functions of these differentially expressed genes and the related biological processes and metabolic pathways in which they participate.

In the liver, we screened out differentially expressed genes that were significantly associated with high and low egg production through strict statistical criteria. Among these genes, *TRIM7* is a member of the tripartite motif (*TRIM*) family. The ubiquitin ligase *TRIM7* has tumor-promoting and tumor-suppressing activities and functions in a variety of biological processes, including innate immunity, ferroptosis regulation, and cell proliferation and migration [48,49,50]. *CASR* (calcium-sensing receptor) is a protein-coding gene that encodes a plasma membrane G protein-coupled receptor that senses changes in the extracellular concentration of calcium ions and plays a key role in maintaining calcium homeostasis [51,52]. These results also suggest that *CASR* may be a multifunctional receptor for calcium, amino acids, and kokumi substances in chickens [53]. Ten genes, including *SPTBN5*, are considered the most promising genes associated with eggshell spots and are related to immune regulation, calcium transport, and phospholipid metabolism [54]. In the chicken genome, *Gal1* is represented by two homologs, *Gal-1A* and *Gal-1B*, which have distinct biochemical properties, tissue expression profiles, and developmental functions [55]. This gene encodes a neuroendocrine peptide that is widely expressed in the central and peripheral nervous systems as well as in the gastrointestinal tract, pancreas, adrenal glands, and genitourinary tract. In the stroma, *IL4I1* and *CCL19* are involved in immune system regulation, such as controlling inflammatory responses and signaling to T and B lymphocytes [56,57]. In the SYF, *CXCL13L2* is a chemokine that guides lymphocyte migration in the immune system and is enriched in the cytokine–cytokine receptor interaction pathway [58,59]. *ZP1* is a protein-coding gene. Research has shown that *ZPC* secreted by granulosa cells specifically binds to *ZP1*, and this phenomenon may be related to the formation of insoluble PL fibers in quail ovaries [60]. Mutations in this gene are responsible for oocyte maturation defects and infertility [61]. Research has shown that chicken *CCL26* could be the major chemokine in chicken adipocytes because of the status of *CCL2* in mammalian adipocytes [62]. *CCR3* acts as a ligand for the C-C chemokine receptor *CCR3*, which triggers Ca^2+^ mobilization in eosinophils [63]. Considering the DEGs and literature review, we selected the *TRIM7*, *CASR*, *SPTBN5*, *GAL1*, *ZP1*, *IL4I1*, and *CCL19* genes as the candidate genes that may be responsible for the differences in egg-laying performance between HEP and LEP chickens. These genes affect egg-laying performance in chickens through different biological processes and molecular functions, especially lipid metabolism, immune regulation, and cell signaling.

A limitation of this study is the relatively small sample size, with 110 chickens analyzed for whole-genome sequencing and 47 samples used for transcriptome analysis. This sample size was suitable for identifying significant genetic variations and differentially expressed genes. However, the limited number of samples may not fully capture the genetic diversity within the Puan Panjiang black-bone chicken population or adequately account for potential environmental factors influencing egg-laying performance. Therefore, the larger sample size and broader population coverage will be crucial for validating these findings and gaining deeper insights into the genetic mechanisms underlying egg production traits in the future.

## 5. Conclusions

This study provides detailed genome and transcriptome analyses of Puan Panjiang black-bone chickens from Guizhou, China, highlighting genetic factors related to egg production. Using a sample of 122 chickens, whole-genome resequencing and differential gene expression analyses identified key genetic variants and genes, such as *TRIM7*, *CASR*, *SPTBN5*, *GAL1*, *ZP1*, *IL4I1*, and *CCL19*. These genes are involved in important biological processes, such as immune regulation, lipid metabolism, calcium transport, and hormonal regulation, which are essential for optimizing egg production. This research reveals the genetic diversity of the Puan Panjiang black-bone chicken and its value as a genetic resource for poultry breeding and advances the understanding of the biological mechanisms behind egg production, supporting the development of breeding strategies to increase productivity and sustainability in poultry industries. This study represents a significant advancement in poultry genetics, assisting in the conservation of local breeds and their utilization in sustainable agriculture.

## Figures and Tables

**Table 1:** Summary of samples and sequencing types.

Groups	Genome Resequencing	Transcriptome Sequencing			
		Liver	Stroma	SWF	SYF
High egg production (HEP)	*n* = 55	*n* = 6	*n* = 6	*n* = 6	*n* = 6
Low egg production (LEP)	*n* = 55	*n* = 6	*n* = 6	*n* = 6	*n* = 5
Red jungle fowl (RJF)	*n* = 17	NA *	NA *	NA *	NA *

* Data from NCBI Genome Database.

**Table 2:** Top ten selected genes in RJF compared to two chicken populations.

RJF vs. HEP		RJF vs. LEP	
Gene	*Fst*	Gene	*Fst*
*ENSGALG00000035230*	0.735898	*TCF25*	0.752545
*KCNQ3*	0.726204	*PSD3*	0.729954
*ENSGALG00000061329*	0.725069	*KCNQ3*	0.723179
*PLAA*	0.72476	*PLAA*	0.711402
*TCF25*	0.687422	*ENSGALG00000000059*	0.679133
*ENSGALG00000003605*	0.686065	*MC1R*	0.679133
*ENSGALG00000013091*	0.667164	*ENSGALG00000061329*	0.654259
*PSD3*	0.652438	*ENSGALG00000035230*	0.643099
*MOG*	0.651331	*BECN1*	0.626708
*SSBP2*	0.640134	*PSME3*	0.626708

**Table 3:** Top five highly differentiated expressed genes in each tissue.

Liver			Stroma		
gene	log2FC	sig	gene	log2FC	sig
*ENSGALG00010005847*	−4.56	down	*ENSGALG00010005680*	−5.10	down
*ATP13A5*	−4.55	down	*ENSGALG00010005332*	−4.03	down
*SPTBN5*	−4.41	down	*ENSGALG00010011634*	−4.00	down
*ENSGALG00010008882*	−4.32	down	*SAA*	−3.79	down
*ENSGALG00010003262*	−4.30	down	*CXCL13*	−3.66	down
*DSC1*	5.29	up	*MHCY44*	4.18	up
*TRIM7.1*	4.92	up	*CHIR1AB4*	4.09	up
*CASR*	4.58	up	*CLPS*	3.66	up
*ENSGALG00010002949*	4.13	up	*ENSGALG00010004778*	3.63	up
*ELAPOR2*	4.11	up	*LOC420808*	3.55	up
**SWF**			**SYF**		
gene	log2FC	sig	gene	log2FC	sig
*ENSGALG00010003757*	−5.22	down	*ENSGALG00010003785*	−4.49	down
*SPIC*	−4.46	down	*SPIC*	−4.44	down
*ENSGALG00010011870*	−4.17	down	*ENSGALG00010003794*	−4.32	down
*LOC121110775*	−4.05	down	*ENSGALG00010003577*	−4.20	down
*ENSGALG00010003477*	−3.97	down	*ENSGALG00010003782*	−4.10	down
*ENSGALG00010000180*	5.80	up	*LOC121113271*	5.20	up
*ENSGALG00010007873*	4.20	up	*ENSGALG00010000180*	5.04	up
*LOC121113271*	3.99	up	*ENSGALG00010002949*	3.60	up
*ENSGALG00010004778*	3.63	up	*ENSGALG00010007873*	3.45	up
*ENSGALG00010000156*	3.59	up	*GAD1*	3.19	up

## Data Availability

The datasets presented in this study are available in online repositories. The repository name and accession number(s) are as follows: PRJNA1100236.

## Supplementary Materials

The following supporting information can be downloaded at: https://www.mdpi.com/article/10.3390/ani15030363/s1, Table S1. Data summary of genome resequencing; Table S2. Categorization of SNPs for HEP and LEP groups; Table S3. Data summary of transcriptome sequencing.
